# A Laboratory Guide in Urinalysis and Toxicology

**Published:** 1887-03

**Authors:** 


**A Laboratory Guide in Urinalysis and Toxicology.** By R. A. WITTHAUS, M. D., Professor of Chemistry and Physics in the Medical Department University City of New York, etc., etc. 57 Lafayette Place: William Wood & Co. 1886.

This little work, in its arrangement, follows Draper's Laboratory Course in Medical Chemistry. Like it, it is a convenient guide to the student, and even physicians will find here the usual tests, so arranged as to be easily and quickly referred to.

